# *Fusarium* Toxins in Cereals: Occurrence, Legislation, Factors Promoting the Appearance and Their Management

**DOI:** 10.3390/molecules21050627

**Published:** 2016-05-13

**Authors:** Davide Ferrigo, Alessandro Raiola, Roberto Causin

**Affiliations:** Department of Land, Environment, Agriculture and Forestry, University of Padua, Campus of Agripolis, Viale Università 16, 35020 Legnaro, Padua, Italy; davide.ferrigo@unipd.it (D.F.); alessandro.raiola@unipd.it (A.R.)

**Keywords:** *Fusarium* toxins, *Fusarium* disease, mycotoxin regulation, mycotoxin management

## Abstract

*Fusarium* diseases of small grain cereals and maize cause significant yield losses worldwide. *Fusarium* infections result in reduced grain yield and contamination with mycotoxins, some of which have a notable impact on human and animal health. Regulations on maximum limits have been established in various countries to protect consumers from the harmful effects of these mycotoxins. Several factors are involved in *Fusarium* disease and mycotoxin occurrence and among them environmental factors and the agronomic practices have been shown to deeply affect mycotoxin contamination in the field. In the present review particular emphasis will be placed on how environmental conditions and stress factors for the crops can affect *Fusarium* infection and mycotoxin production, with the aim to provide useful knowledge to develop strategies to prevent mycotoxin accumulation in cereals.

## 1. Mycotoxigenic *Fusarium* and *Fusarium*-Related Diseases

*Fusarium* is one of the most economically important genera of phytopathogenic fungi. Several *Fusarium* species can infect small grain cereals (wheat, barley and oat) and maize; the predominant species can vary according to crop species involved, geographic region and environmental conditions [1,2]. *Fusarium* toxins are secondary metabolites produced by toxigenic fungi that naturally contaminate cereals, they represent a source of grave concern in cereals and cereal-based products, resulting in harmful contamination of foods and feedstuffs [3].

*Fusarium* diseases that affect cereal crops are caused by several individual *Fusarium* or more commonly, co-occurring species. *Fusarium* spp. can cause indirect losses resulting from seedling blight or reduced seed germination, or direct losses such as seedling foot and stalk rots; however, the most important diseases in cereals due to a severe reduction in yield and quality are head blight of small cereals as wheat, barley and oat, and ear rot of maize [4,5]. The coexistence of different *Fusarium* spp. in the field is a normal situation and although the number of detectable species can be high [6], only some of them are pathogenic, especially under suitable climatic conditions. The composition of species involved in the *Fusarium* disease complex is dynamic [7]. The species comprising a *Fusarium* community associate with each other and this cohabitation is particularly affected by climatic factors such as temperature and moisture. Moreover, evidences indicates that the environmental conditions that favour the infection process can differ from those that affect colonization [8]; therefore, the relationship among *Fusarium* species may change over time during the infection process.

*Fusarium* head blight (FHB) of small grain cereals is associated with up to 17 *Fusarium* species [9], but only a few of them are important worldwide in terms of diffusion and economic impact. Moreover, under cool and wet conditions, *Microdochium nivale* (syn. *Fusarium nivale*) represents an important co-occurring causal agent of FHB. The environmental conditions that promote FHB are moderate temperatures in the presence of high humidity. In addition, FHB is favoured by rainfall during and after flowering. The two main species responsible for FHB are *Fusarium graminearum*, a dominant species in warm and wet conditions, and *Fusarium poae*, which occurs under relatively warm and dry conditions [10,11]. *F. graminearum*, along with at least 16 different species belonging to the *F. graminearum* complex (FGC) [12], is the most prevalent and aggressive causal agent of FHB on both wheat and barley worldwide [13,14]. *F. graminearum* is prevalent in southern Europe [15], America [16] and Asia [17], however, it has been found to spread toward the cooler regions of Europe: Finland, Russia and Sweden [18,19]. Although *F. poae* has been previously considered a relatively low virulent pathogen of cereals compared with *F. graminearum*, recent studies identified this pathogen as the major FHB component of wheat in certain years or in different geographical areas [20]. The variability in the predominance of *F. poae* is highly influenced by the climate conditions of locations that are characterized by cold and moisture, where this fungus presents a high frequency but a lower density compared with warmer areas. This suggests the greater adaptability of *F. poae* under certain agro-environmental conditions where other *Fusarium* spp., such as *F. graminearum*, are less favoured. Other species such as *F. avenaceum*, *F. culmorum*, *F. sporotrichioides* and *F. langsethiae* are FHB agents considered to be of secondary importance; however, they can play significant roles in pathogenesis when climatic conditions are not favourable for the development of the main FHB causal agents [18,21,22].

In maize, *Fusarium* infection of the ear and kernels comprises two distinct diseases: *Gibberella* ear rot (GER) or “red ear rot” prevalently caused by species of the *Discolor* section, and *Fusarium* ear rot (FER) or “pink ear rot”, mainly caused by species of the *Liseola* section [5]. Similar to FHB in wheat, *Fusarium* diseases in maize are characterized by the co-presence or rapid succession of different species; furthermore, GER and FER may be present on the ears at the same time. The prevalence of ear rot type varies according to the causal species, which mainly depends on the climatic parameters, the agronomic practices, the local composition of the fungal community and the susceptibility of the host plant. *Fusarium* diseases in maize are also influenced by insect activities that result in injured kernels. In fact, species belonging to the *Liseola* section are prevalent on ears damaged by corn borers whereas *Fusarium* belonging to *Discolor* section are well represented on maize ears that are not damaged by insects [23]. This behaviour appears to be particularly linked to the different ways in which *Fusarium* colonizes ears; in fact, *F. verticillioides*, which is characterized by low virulence, typically infects plants through injuries [24] and in lesser extent silks [25], while *F. graminearum* is highly virulent and can strongly infect plant tissues [26]. The predominant species causing GER are *F. graminearum*, *F. culmorum* and to a lesser extent, *F. avenaceum*, however, several other species such as *F. equiseti*, *F. poae*, *F. sporotrichioides*, *F. acuminatum*, *F. semitectum*, *F. solani* and *F. temperatum* can be isolated with lower frequency from molded maize ears. The dynamic of infection and fungal community involved in GER follow the same behaviour observed in FHB of small cereals and is favoured by high moisture at silking under warm conditions [27,28].

The main *Fusarium* species involved in FER is *F. verticillioides*, with a 100% incidence under conducive conditions; however, also *F. proliferatum* and *F. subglutinans* are important causal agents. *F. verticillioides* is likely the most common species isolated worldwide from diseased maize [29]. Compared with GER, FER occurs under hotter and drier conditions, especially after pollination [30]. The predominance of *F. verticillioides* among *Fusarium* isolates has been observed in Europe [31,32], Africa [33], Asia [34] and America [35,36] over time, with a colonization incidence of up to 90%. *F. verticillioides* is often associated with *F. subglutinans*, which occupies the same ecological niche and thus competes for nutrients and space. In addition, the incidence of *F. proliferatum* populations in southern maize-growing areas has been widely reported [37]. The dynamics of fungal communities involved in FER are strongly influenced by interactions with host and environmental factors, in fact *F. subglutinans* and *F. proliferatum* occur as moderately aggressive pathogens but are generally considered to require cooler temperatures than *F. verticillioides* [38], which is characterized by low pathogenicity [39,40] but high adaptability to the hot conditions.

Although cereal fusariosis strongly affects crop production, several *Fusarium* spp. also produce a wide range of toxins that can reach concentrations harmful to humans and animals. The particular mycotoxin combination is species- [41,42] and strain-specific [43,44]; moreover, the toxigenic profile of a contaminated crop is determined not only by the predominant pathogenic species but also by the lesser species in the *Fusarium* community.

## 2. *Fusarium* Mycotoxins

**Figure 1:**
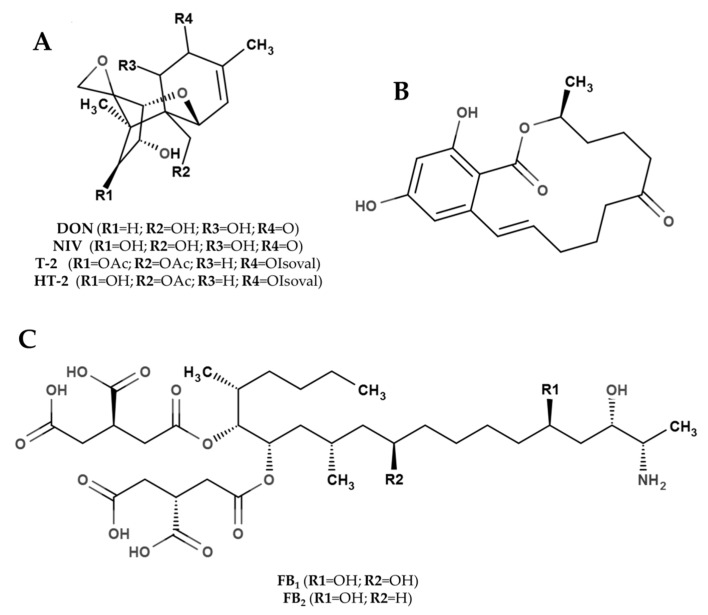
Chemical structure of the main *Fusarium* mycotoxins. (**A**) Trichothecenes; (**B**) Zearalenone; (**C**) Fumonisins; OAc = acetyl function; OIsoval = isovalerate function.

The most common *Fusarium* mycotoxin groups are trichothecenes, zearalenones and fumonisins (Figure 1); however, other mycotoxins (enniatins, moniliformin, beauvericin and fusaproliferin) can be identified in combination with the above-mentioned toxins [45].

The recently published levels of mycotoxin contamination in main countries and selected regions of the world, according to the importance of the amount of cereals produced are presented, in Table 1, Table 2, Table 3 and Table 4, below. Because no recent data were found on the overall mycotoxin contamination in different countries, what is shown here are the mycotoxin levels in different geographical areas, which, although not fully representative, can provide a comparison term between the measured contents and the stringent European normative. For this reason the tables show, where present, the percentage of samples that exceed European limits.

The distribution of mycotoxins in different regions is determined not only by the environmental conditions that affect *Fusarium* populations but also by endogenous and exogenous factors that can affect mycotoxin production.

### 2.1. Trichothecenes

Trichothecenes (Figure 1A) comprise a vast group of metabolites containing an epoxide, which is responsible for their toxicological activity. Trichothecenes produced by *Fusarium* spp. are widespread in all cereal-growing areas of the world and they are divided into two groups: A and B, mainly characterized by the presence of different functional groups in the C-8 position of the trichothecene backbone [71]. The A group mainly includes T-2 and HT-2 toxins, diacetoxy- and monoacetoxy-scirpenol (DAS and MAS) and neosolaniol (NEO). The B group mainly includes deoxynivalenol (DON), nivalenol (NIV), 3-AcetylDON, 15-AcetylDON and fusarenone X [72,73]. *Fusarium* *langsethiae*, *F. equiseti*, *F. poae*, and *F. sporotrichoides* produce type A trichothecenes while *F. culmorum* and *F. graminearum* typically produce type B trichothecenes. However, recently identified strains of *F. graminearum* are able to produce in wheat new trichothecenes (named NXs) with a structure similar to the type A [74]. Therefore, a strict separation among types of trichothecenes belonging to specific *Fusarium* species does not appear to be useful for systematic separation.

DON and NIV are the main type B trichothecenes found in *Fusarium*-infected kernels. A worldwide prevalence of DON-producers *Fusarium graminearum* complex species is known [75]; however, the occurrence of populations with high predominance of NIV-producers has been reported [76]. Deoxynivalenol (DON), also known as vomitoxin, is the most frequently occurring trichothecene in small cereals and maize used for food and feed production [77]. DON can also be present as mono-acetylated (3-AcDON, 15-AcDON) and di-acetylated (3,15-AcDON) derivatives [37]. DON has the potential to cause chronic effects such as reduced growth and anorexia, as well as acute intoxication leading to vomiting (emesis), immunotoxic effects and changes in brain neurochemicals [78]. In pigs, DON is also implicated in reproductive disorders with direct effects on ovarian function [79] and similar effect are presumed in cattle [80].

Nivalenol (NIV) is one of the well-known type B trichothecenes and usually occurs with other toxins among which DON, DAS and T2 [81,82]. With respect to the harmfulness of toxins, NIV is more toxic than DON towards animals [83] while DON is more toxic against plants [73]. NIV is a potent inhibitor of protein, RNA and DNA synthesis in mammalian cells and can cause necrosis of cells, especially in tissues that are rapidly growing and dividing as intestinal epithelial cells [84,85]. NIV can be present as di-acetylated derivatives, *i.e.*, 4,15-AcNIV, produced by some strains of *F. graminearum*, *F. cerealis*, *F. poae* and *F. culmorum* [86]. Due to the ecology of the main producing species, NIV has usually been reported in cereal during years that are characterized by relatively drier and warmer growing seasons (with respect to DON) [87]. Generally, NIV contamination of cereals appears to be lower than DON [57,88], the low exposures to nivalenol, based on the available occurrence data in food, led EFSA to consider nivalenol not a health concern [89].

T-2 and HT-2 toxins are type A trichothecenes produced by several *Fusarium* spp., mainly *Fusarium* *langsethiae*, *F. sporotrichioides* and *F. poae* in small grains and are most commonly present in oat. *F. langsethiae* and *F. sporotrichioides* are considered to be the main producers of T-2 and HT-2 toxins, especially in Northern Europe [90,91]. T-2 is rapidly metabolized *in vivo* to HT-2, which induces adverse effects similar to T-2, with non-remarkable differences in terms of strength [92]. Being a potent inducer of oxidative stress and an inhibitors of DNA, RNA, protein synthesis and mitochondrial function, T-2 and HT-2 toxins represent contaminants that are of considerable concern for human and animal health [93,94]. Furthermore, T-2 and HT-2 contamination can occur with diacetoxyscirpenol (DAS) [41,95], that is expectable because DAS is biosynthesized at a side branch of the T-2 toxin pathway [96].

### 2.2. Zearalenone

Zearalenone (ZEA, Figure 1B), a phenolic resorcylic acid lactone, is a mycotoxin that may occur in the form of four hydroxyl derivatives [3]. ZEA is of major interest because despite its low acute toxicity, it has proven to be hepatotoxic, immunotoxic, and carcinogenic to a number of mammalian species [97]. Moreover, ZEA and some of its metabolites have been shown to competitively bind to estrogen receptors in a number of different species and are responsible for hyper-estrogenism and infertility in livestock [98]. ZEA is mainly produced by *F. graminearum*, *F. culmorum*, *F. cerealis*, *F. equiseti* and *F. semitectum* [99] and the contamination often co-occurs with DON. It is a common *Fusarium* mycotoxins in the temperate regions of America [47], Europe [100] and Asia [101], but also present in Africa [63]. This toxin has a worldwide distribution with differences in the percentage and level of contamination, which are generally lower compared with the most representative trichothecenes (DON) [102].

### 2.3. Fumonisins

Fumonisins (Figure 1C) are a group of polyketide-derived mycotoxins that have a wide geographic distribution, and are consequently most commonly present on maize in many different regions [103]. Although up to 13 *Fusarium* species are able to produce fumonisins [104], *F. verticillioides* and *F. proliferatum* are the most important species associated with fumonisin contamination. Fumonisins can cause severe disorders in animals [105], apoptosis as consequences of membrane lipid peroxidation [106]. Consumption of fumonisin-contaminated maize has been associated with esophageal cancer and embryonal neural tube defects in humans [107]. Fumonisins can be separated into four main groups, identified as the fumonisin A, B, C, and P series [108]; the B group includes the most active fumonisins FB_1_ and its isomers FB_2_, FB_3_ and FB_4_ [109]. In particular, FB_1_, which causes considerable toxicological concern, is the most abundant fumonisin produced in maize. FB_1_ accounts for 70%–80% of total fumonisins compared with 15%–25% (FB_2_), 3%–8% (FB_3_) and 1%–2% (FB_4_) [110]. Fumonisin contamination of cereals is a worldwide concern, and *F. verticillioides* is the main fumonisin producer. In regions characterized by temperate-warm conditions, a high incidence of fumonisin contamination is usually present [111].

On the base of differences in the levels of mycotoxin contamination that can be observed worldwide, it appears evident that in different environments and in particular when the weather conditions are unfavorable or in absence of appropriate management the levels of contamination can exceed the maximum and tolerable limits. So, the adoption of forecast models and appropriate management strategies at the production stages to contain mycotoxin occurrence appears encouraged. The different regulations on mycotoxin levels must be considered in view of a global market and since European regulations appear stringent, a common strategy looks like the best way for food safety.

### 2.4. Emerging Fusarium Toxins

Beyond to the most common *Fusarium* toxins, others considered emerging have been reported in huge quantities and the contamination seems to be related to climate condition and cereal type. Among the emerging mycotoxins, enniatins, beauvericin, fusaproliferin and moniliformin represent a potential health treat to investigate.

Enniatins (ENs) and beauvericin (BEA) are mycotoxins produced by several *Fusarium* species that are known to contaminate cereals and by-products [112,113]. These toxins show similar chemical structures and present the same toxic dynamic actions, exhibiting antibiotic, antimicrobial, insecticidal and cytotoxic effects. Further, they are easily incorporated into cellular membranes, disturbing the physiological ionic balance, which affects cell homeostasis [114]. For several cell lines, cytotoxicity of these mycotoxins has been demonstrated to inhibits cell proliferation modifying cell cycle phases and increasing apoptosis induce apoptosis and mitochondrial damage [115].

ENs are six-membered cyclic depsipeptides, consisting of at least 23 different compounds that have been described as naturally occurring enniatin analogues [116,117]. However, the more common analogues are the enniatins B_1_ and A_1_. Enniatin occurrence is typically high in Northern and Eastern Europe barley and wheat [57,118] with incidence up to 100%, but also Mediterranean climate can favors the growth of toxigenic molds that produce ENs. An analysis conducted on cereals from Spain indicated that frequencies of EN contamination were 89%, 62% and 50% for maize, wheat and barley, respectively [119]. Similar high incidences were found in Morocco [120] and Tunisia [121], although at low levels.

BEA is a mycotoxin of the cyclohexadepsipeptide family. It has been found as a natural contaminant of cereal in Europe [122], Africa [123], America [48] and Asia [124]. *Fusarium* *poae*, mainly, but also *F. avenaceum*, *F. temperatum* and many others have been described as producers of BEA [125,126]. BEA was found to be present in 26.5% of Moroccan cereals samples with a maximum contamination in maize [120], while low contamination was found in cereals from Portugal [127] and Mexico [128]. In Argentina, potentially contaminated maize samples were observed [126] while no contamination was reported for wheat [129].

Fusaproliferin (FUS) is a bicyclic sesterterpene produced by Fusarium proliferatum, *F. subglutinans*, *F. antophilum*, *F. begoniae*, *F. bulbicola*, *F. circinatum*, *F. pseudocircinatum*, *F. guttiforme*, *F. concentricum*, *F. succisae*, *F. udum* [130] and *F. temperatum* [126]. FUS can usually be found in a deacetylated form in a 3:1 ratio [131]; however, the deacetylated form shows a limited toxicological activity compared with FUS [132]. This compound is toxic to brine shrimp (*Artemia salina* L.), insect cells and mammalian cells, and causes teratogenic effects in chicken embryos [130,132,133]. The production of FUS by F. proliferatum strains and the natural co-occurrence of these mycotoxins in maize samples contaminated by Fusarium species have been reported in Italy, South Africa and USA [132,134,135]. Data indicated weak FUS contamination levels in cereals from Morocco [120], but no contamination was observed for samples from Tunisia [121]. FUS was very common in maize in Mexico [128], but at very low levels, while it was not detected in wheat in Argentina [129].

Moniliformin (MON) is a small and highly polar molecule present in nature as a water-soluble sodium or potassium salt [136]. *Fusarium* species most frequently associated with MON production throughout the world are *F. proliferatum*, *F. verticillioides*, *F. subglutinans*, *F. avenaceum*, *F. chlamydosporum*, *F. oxysporum* and *F. tricinctum* [1,137]. *Fusarium* *avenaceum* appears to be the most important producer of moniliformin (MON) and ENs, at least in the Nordic countries [45].

The molecular mechanism of MON action is unknown; however, because of its structural similarity to pyruvate, MON probably affects metabolic pathways involving pyruvate and the inhibition of the oxidation of tricarboxylic acid (TCA) cycle intermediates, resulting in respiratory stress [138]. The occurrence of moniliformin in cereals and cereal products has been reported for different regions worldwide [139], and variable levels have been recorded. Recently, levels up to 2500 µg/kg were reported in maize samples from northern Italian fields [140], with an overall incidence of 93% positive samples. High MON levels were also find in Nordic wheat and maize, with lower levels in barley and oats [141,142]. MON contamination of Canadian durum wheat, soft wheat, rye and oat samples was 75%, 56%, 33% and 16%, respectively [48].

## 3. Legislation on *Fusarium* Toxins in Cereal

Mycotoxins are one of the most important risks associated to cereals consumption [143] and in some cases they may also have a negative impact on the quality of the food and feed. To preserve the public health and livestock production by mycotoxin occurrence the countries developed measures as the introduction of maximum or recommended levels for food and feedingstuff.

Internationally, Codex Aliment Arius Commission (CAC) issues legislation on food and feedstuff. The CAC, established by World Health Organization (WHO) and Food and Agriculture Organization (FAO), has issued international standards, guidelines and codes of practice for the prevention and reduction of mycotoxin contamination in several foods and feeds; in CAC/RCP 51-2003 document are included *Fusarium* toxins in cereals [144].

Among factors that play a major role on defining limits and regulations for mycotoxins are included the availability of toxicological and exposure data, the knowledge of the distribution of mycotoxin concentrations in commodity and these limits are provided for mycotoxin/matrix combination [145]. The CAC has recently proposed a draft of the maximum limits for human consumption of DON in raw wheat, maize, barley grains and their derivatives at 2000 and 1000 ppb, respectively; fumonisins in unprocessed maize grain and derivatives at 4000 and 2000 ppb, respectively [146].

Specific regulations at country level are proclaimed by authoritative bodies, for example, European Commission, Food and Drug Administration of United States (U.S. FDA), Public Health Agency of Canada (PHAC), Health Surveillance Agency for Brazil (ANVISA), Food and Drug Administration of China (CFDA) and the Russian Federal Service for Surveillance on Consumer Rights Protection and Human Wellbeing (Rospotrebnadzor).

Due to the heterogeneity of commodities produced and consumed, the European regulation on mycotoxins is probably the most complete, comprising the majority of contaminant toxins; therefore the comparison of limits of *Fusarium* toxin was performed on the base of European levels. In Table 5 are listed the limits established by authoritative bodies for mycotoxin contamination in Europe. Despite the great worldwide production of rice, this cereal will not discussed in this review given the low contamination levels of *Fusarium* toxins in polished products for human consumption compared to the other small cereals [147,148]. Over the years, the number of countries with known specific mycotoxin legislation has increased with particular emphasis to the main food and feed cereal commodities produced or traded by a specific country. Outside Europe, in the main cereal producing countries, e.g. in Brazil, *Fusarium* mycotoxin regulation, in effect since 2016, indicates the maximum tolerable limits for deoxynivalenol in whole wheat and wheat derivatives at 1000 and 750 ppb, respectively. About zearalenone the maximum tolerable limits in whole wheat, wheat flour and derivatives, maize and derivatives are 200, 100 and 150 ppb, respectively. About fumonisins the maximum tolerable limits in maize meal and other maize-based products are 1500 and 1000 ppb, respectively [149].

In Canada, *Fusarium* mycotoxin regulation in food establishes tolerable level of deoxynivalenol in uncleaned soft wheat at 2000 ppb (under review) [150]. In China, *Fusarium* mycotoxin regulation in food establishes the maximum level for deoxynivalenol in wheat, barley, maize and derivatives at 1000 ppb; about zearalenone the maximum levels in wheat and maize are 60 ppb [151]. In Russia, *Fusarium* mycotoxin regulation in food establishes the maximum level for deoxynivalenol in wheat, barley and their derivatives at 700 and 1000 ppb, respectively. About T2 in food grain and their derivatives limit is 100 ppb; about zearalenone in wheat, barley and maize maximum levels is 1000 ppb. About fumonisin in maize flour permissible levels are not more than 200 ppb [152]. Finally, the U.S. Food and Drug Administration (FDA) recommends that DON levels in human foods should not exceed 1000 ppb. About fumonisins in degermed dry milled corn products and cleaned maize used for popcorn limit levels are 2000 and 3000 ppb, respectively [153,154].

As previously reported, toxicosis in animal fed with feedstuff contaminated by Fusarium toxins led to a worsening in animal productivity and general healthiness, resulting in increased susceptibility to parasites and diseases. To reduce issues related to mycotoxin occurrence in feedstuff, legislation ruled the presence of these compounds in products intended for animal feeding. For an illustrative purpose, in Table 6 are listed the European recommended guidance values relative to feedstuffs.

## 4. Factors affecting *Fusarium* Toxins Production

### 4.1. Effect of Climate Events on FHB, Maize ear Rots and Mycotoxin

Climate is among the most important factors influencing the occurrence and distribution of *Fusarium*. Different climatic conditions (e.g., temperature and rainfall) in different geographical locations affect the incidence of pathogens responsible for FHB of small grain cereals and ear rots of maize. The relationships between climatic factors and FHB development have been thoroughly investigated [14,157]. Well define ranges of temperature and water availability are determining factors for the growth of *Fusarium* and mycotoxin production [42]. Warm and moist conditions, especially during the period of anthesis, are considered critical factors for FHB development. Among the FHB causal agents, the fungal species vary on a regional and continental scale and during any given season [158,159]. It is conceivable to suggest that under the influence of climatic changes, modifications in the total and relative abundance of fungal species of the FHB complex may occur. Changes in climatic extremes would have direct impacts on *Fusarium* ear disease and mycotoxin production because weather factors can strongly affect epidemics and the proportions of the species responsible for FHB and ear rots [160]. These changes could also influence the production of DON by the two main DON-producing fungal species, *F. graminearum* and *F. culmorum*, as well as the production of fumonisins by the main producer, *i.e.*, *F. verticillioides*. In fact, temperature that may be optimal for growth, are different from those optimal for mycotoxin synthesis by *F. graminearum*, *F. culmorum* and *F. verticillioides* [161,162].

In maize, climatic factors determine the balances that occur within *Fusarium* populations. Maize ear rots are caused by a mixture of pathogens that compete among themselves. It is generally recognized that negative interactions in competition between *Fusarium* spp. are prevalent. GER and FER are favoured by distinct climatic conditions: GER is favoured by high levels of moisture at silking, followed by moderate temperatures and high rainfall during the maturation period [163] while FER is more common in warmer and drier areas [37]. In fact, *F. verticillioides* prefers a higher temperature of 30 °C and tolerates water stress better than *F. graminearum*. Fumonisin contamination is highly dependent on the composition of the *Fusarium* community as well as environmental conditions, and fumonisin incidence can be high or low in relation to the growing areas. Several evidence indicates that water stress during drought events is strongly associated with high levels of *F. verticillioides* infection and fumonisin accumulation in kernels [27]. In particular, the factors that affect fumonisin development include environmental factors (temperature, humidity), insect damage and pre-/post-harvest management.

More, *F. verticillioides* isolates were found to exhibit better performance at higher temperatures and under water stress conditions in comparison to *F. proliferatum*, another fumonisin producing species [164]. The levels of FB_1_, the most abundant and toxicologically active fumonisin, were found to be absent or significantly low in areas generally characterized by cold and wet seasons [165]; however, under favourable conditions in these areas, fumonisins reached significant levels [166].

### 4.2. Fungal Interactions in Cereals: Consequences for Fusarium Development and Mycotoxin

Interactions among fungal species depend on biotic and abiotic factors and can play an important role in the structural organization of fungal communities. These interactions range from antagonistic to mutualistic and can be positive, negative or neutral [167]. Through different mechanisms (competition for space and feed resources), some pathogenic species may have an advantage over other fungal species that occupy the same niche, hindering the development of less competitive fungi [168]; on the contrary, one fungal species can improve the adaptability of other species [169]. Therefore, the role of ecological interactions is of particular importance because these interactions can significantly affect fungal development and secondary metabolism. It is critical to take this into account to accurately assess the risk of mycotoxin contamination. Moreover, host-specific influences on intraspecific competition may dictate fungal compositions and probably mycotoxin occurrence as observed in *A. flavus* populations [170]. Several studies on interspecific interactions between only *Fusarium* spp. or between *Fusarium* spp. and other genera have been carried out under *in vitro* conditions [171,172], whereas only a few have been conducted under natural conditions [173] Negative interactions in fungal communities that occupy the same ecological niche are predominant and are based principally on competition [174,175].

In small grain cereals, FHB is generally associated with various fungal species, including both toxigenic (several species of *Fusarium*) and non-toxigenic fungi (*Microdochium* spp.), and their prevalence and abundance in the same field [176,177] are strongly dependent on environmental variables. Due to environmental variables under field conditions, FHB development and mycotoxin production are predicted to be more complex when more than one toxigenic species is present. Also the role of conidia has been evaluated and studies on interactions between several FHB species have shown that among *Fusaria* the species producing macroconidia are the most competitive during germination [178]. Experimental evidence supports any synergetic interactions between single isolates of *F. graminearum*, *F. poae*, *F. culmorum* and *F. avenaceum* after inoculation on wheat spikes, while in most cases, the presence of competitive interactions is more evident. In the presence of a mixed FHB infection, a large reduction in fungal biomass has been observed in comparison to single inoculations. On the contrary, mycotoxin productivity per unit of fungal biomass was found to increase dramatically in the co-inoculations, indicating that the production of trichothecene mycotoxins can be affected by competition [173]. Some experimental studies performed in wheat do not confirm these results where intraspecies interaction appears to reduce trichothecene yield [179]. Recent findings suggest that the behaviour of different isolates in presence of a competitor is variable mostly depending by *Fusarium* strain rather than species, with a predominance of aggressive isolates [180]. However, this study also demonstrates a lack of correlation between co-occurrence of several FHB species and an increase of *Fusarium* toxins risk in wheat production.

In maize, in competition with other genera that commonly co-occur on kernels, *F. verticillioides* has been found to take advantage of *Aspergillus flavus* and *Penicillium* spp. in mixed infections [181,182]. Towards other *Fusarium* species, inoculation of maize with isolates of *F. verticillioides, F. proliferatum* (fumonisin producers) and *F. graminearum* (DON and zearalenone producer), performed under different water and temperature conditions, showed that *Fusarium* populations generally decreased in presence of competitors in dependence of environmental variables. In addition, fumonisin production was generally reduced in competing interactions, whereas zearalenone was not affected and DON was increased [183]. These *in vitro* experiments indicated opposite results compared with those obtained from trials under natural conditions where *F. verticillioides* has been observed to inhibit the growth of *F. graminearum* [167]. It is known that *F. verticillioides* has a competitive advantage over *F. graminearum* when simultaneously inoculated due to better growth and a higher spore germination rate over a wider range of temperatures and water activities [167]. However, the impact of these interactions on mycotoxin contamination requires further investigation with respect to environmental and stress conditions. Indeed, it was demonstrated that high levels of *F. verticillioides* do not necessarily result in high levels of fumonisin contamination [184]. Insensitivity of ZEA and DON producers to competition also occurred when *F. graminearum* was cultivated with *Aspergillus parasiticus*, and the toxin levels were not modified [185].

### 4.3. Stress Factors

Depending on their environmental growth conditions, fungi sense a variety of external signals and respond by regulating secondary metabolism [186]. Field crops are continuously challenged by several environmental stresses that occur naturally in a certain area. Cereal growth, productivity and resistance to pathogens are closely related to environmental and agronomical input, which are both related to the response of crop plants to stress. Stress conditions imposed on developing crops, especially during the reproductive stage, can facilitate fungal infection, mycotoxin production and grain contamination [187].

Biotic factors such as insects, pathogens and weeds [188,189,190] and abiotic factors such as hot temperatures, drought and hailstorms [191] can affect crop physiology and productivity [192] and may result in conditions that are favourable for mycotoxin accumulation. There is evidence that the abiotic and biotic factors that predispose plants to diseases can activate several plant responses to stress, which can indirectly influence mycotoxin production [193]. In response to biotic and abiotic stress, plants react with a rapid and transient release of reactive oxygen species (ROS), activating a broad range of strategies to protect themselves [194,195,196]. Because oxidative stress in fungi was demonstrated to modulate *in vitro* the biosynthetic pathways of *Fusarium* mycotoxins such as trichothecenes and fumonisins [197,198], it is conceivable to suggest that an alteration of the cellular redox state *in planta* can affect mycotoxin accumulation. 

One of the primary biotic stress factors that influence fungal colonization and mycotoxin contamination are the insects. As a consequence of phytophagous insect attack , the harmful action of insects occurs in two ways: by producing wounds that are favourable entry sites for conidia already present on the ear tissues and by causing stress conditions in plant tissues [199] through the generation of ROS [200]. ROS generation in plant was suggested to be a common response that persists on as long as the insect attack carries on. Although the effect of insect activity on small cereal is low, insects can still be considered a potential risk for the occurrence of FHB. In fact, pre-exposal of wheat ears to aphids as *Rhopalosiphum padi* and *Sitobion avenae*, can co-occur with FHB appearance and lead to a significant increase in *F. graminearum* colonization and DON accumulation [201,202]. These results are probably related to the elicitation of defense signalling pathways through accumulation of H_2_O_2_ and ROS [203] as well as enhancement of plant defence [202]. As regards to the redox potential, this can act as a modulator of DON biosynthesis [204] that, in turn, lead to a further accumulation of H_2_O_2_ in wheat tissues [205]. Recently, in *F. graminearum* the gene FGK3, recognized as an important virulence factor essential for pathogenicity and DON production, was demonstrated to be up-regulated in response to H_2_O_2_, cold and SDS stresses [206]. Concerning plant defence, the pathogen can produce more DON in an attempt to circumvent the enhanced defences, with the consequent acceleration of disease progression and mycotoxin accumulation [207].

In maize, *F. verticillioides* infection is facilitated by insect damage while *F. graminearum* mainly infect ear through the silks, therefore, the effect of insect stress is higher for FER pathogens respect to GER ones [5]. Recent evidence has also shown a correlation between ear-feeding insects and mycotoxin contamination in maize [193,208,209], and other findings suggest that kernel-feeding insects are more important than silk- or cob-feeding insects [210]. Insects affecting maize such as *Ostrinia nubilalis* (European corn borer; ECB), *Sesamia nonagrioides* (Mediterranean corn borer), *Helicoverpa zea* (Corn earworm), and *Sitotroga* *cerealella* (Angoumois grain moth) can produce tunnels into stalks and ears and can carry *F. verticillioides* conidia and therefore transmit infection. Beyond to corn borers, also populations of ear-feeding insects as *Frankliniella occidentalis* (Western flower thrips) provide inoculum sites for *Fusarium* spp. and their presence are strongly correlated with disease severity and fumonisin contamination [24].

While it is difficult to distinguish the role of these actions in the fungal infection process and mycotoxin induction, at least for aflatoxin accumulation, it was reported that *Aspergillus flavus* infection mediated by a vector was more conducive than that mechanically mediated, demonstrating the importance of insects in mycotoxin occurrence [211]. Because of the increased level of ROS reported after insect attack on Lima bean and potato [212,213] also the interaction between maize and ECB should represent a stress condition; however, how this stress relates to mycotoxin induction requires further investigation.

The results from several studies suggest a role of ROS such as H_2_O_2_ in mycotoxin production by toxigenic *Fusarium*, as well as antioxidant compounds have been demonstrated to inhibit toxinogenesis [198,214]. The concomitance of multiple pathogens can positively influence *Fusarium* disease in cereals. In maize, the infection by ear-damaging pathogens as *F. graminearum* and *F. subglutinans* facilitates the subsequent *F. verticillioides* infection and fumonisin accumulation [215]. Further evidences are provided by infection of maize with *Ustilago maydis* where fumonisin levels resulted increased in the kernels harvested from smutted ears compared with the kernels from smut-free control ears [189]. Together, these data show that an initial infection can breach the host defense and weaken plants, allowing access to other pathogens, including toxigenic fungi, and promoting their performance.

Weeds represent a threat to the crop and also an indirect stress affecting the crop performance. As reported in maize, light competition with the perennial ryegrass *Lolium perenne* unfavourably modifies the pattern of plant growth and development. This interaction also highlighted as a first stress due to shade avoidance may affect sensitivity toward a subsequent abiotic stress [216]. Moreover, the light reflected from the tissues of the above-ground neighbouring weeds was found to reduce total root biomass [190], furtherly influencing ability of plant to adsorb water and nutrients. Finally, competition between crop plants and weeds for water, nutrients and sunlight involves the ability of a plant to respond to diseases and parasites and crop competition with weeds is presumed to increase sensitivity to soil-borne mutualists and pathogens [217].

Abiotic stress, such as hot temperatures and drought conditions, strongly alters the efficiency of photosystems and the stability of membranes, and is associated with oxidative stress in plants [196]. In fact, crop resistance to stress conditions can be related to high efficiency ROS-scavenging systems, as has been reported in wheat [218]. Because dry conditions typically accompany excessive heat, it is difficult to determine the influence of single factors. During kernel filling, drought and high temperature are considered as the environmental conditions that are most conducive to mycotoxin contamination in maize [219]. In Poland, it was reported that particularly high concentrations of fumonisins were associated with the hottest and driest summers [220]; however, other studies reported a low [24] or no influence of drought stress [221]. Moreover, during field trials conducted in Italy under climatic conditions that were considered unfavourable for *Fusarium* infections, a decrease in *F. verticillioides* colonization in maize was not related to an equal reduction in fumonisin accumulation. This result further suggests that hot conditions and drought stress play an important role in modulation of fumonisin production [184]. Environmentally damaging conditions such as hailstorms have also been reported to decrease quality [222] and increase mycotoxin contamination [191], favouring the entry of a fungal pathogen and causing plant stress. These authors reported that fumonisins were more frequently detected in grain from hail-damaged fields compared with undamaged fields. While an increase in the level of stress signalling following mechanical damage of leaf tissues has been well documented [223], the possibility that this type of meteorological event can stimulate mycotoxin biosynthesis through wound signals should be considered.

## 5. *Fusarium* Disease and Toxins Management

Good Agricultural Practices (GAPs) in cereals provides the adoption of measures in all phases of crop production able to interfere with the *Fusarium* spp. infection and toxins accumulation in grain. The agriculture practices, below described, can differently affect the levels of contamination of the different kind of toxins in maize and wheat. GAPs guidelines have been proposed to the Italian Ministry of Agricultural, Food and Forestry Policies [224], their importance and impact on the main mycotoxins is summarized in Table 7.

### 5.1. Tillage and Crop Rotation

Infected cereal debris, which are major sources of inoculum for *Fusarium* infection [225,226], decompose slowly [227] and can therefore be present in subsequent crops for at least two years [228,229]. With respect to tillage, conventional practices include to plough the soil and bury the remains of previous crops and weeds whereas in minimum or no tillage practices, seeds are directly drilled into the previous crop stubbles.

It is clear that conventional tillage systems alter, with different degrees for a limited time, the physical and chemical property of soil influencing the nutrient distribution and the organism microenvironment, altering microbial population, complexity and layer distribution [230] and these changes are likely influenced by soil structure and environment [231]. No-till management that avoid soil disturbance and increase organic matter modify the microbiota components favouring fungi as primary decomposers with respect to the bacteria [230]. Since complex indigenous fungal communities in arable soil, due to improvement in competition and antagonism, were linked to a role in suppression of soilborne pathogenic fungi as *Fusarium* spp. [232], no-till practices should represent the strategy to counteract *Fusarium* soilborne inoculum.

Some studies, instead, evidenced any effect due to tillage systems for DON level in wheat [233], and also a lack of effects with respect to fumonisin in maize [234]. Furthermore, other authors reported that minimally prepared soil after a *Fusarium*-host crop was conducive to a high incidence of *Fusarium* disease and mycotoxin contamination of wheat and maize [235,236], while any effect was detected when the previous crop was not a *Fusarium*-host plant [237]. These contrasting results suggest the crucial importance of agronomic and environmental factors that can vary in the years and areas in which each trial was carried out.

Although some authors consider adjacent crops as the main source of inoculum [238], others state that due to long-distance transport of viable spores of *Gibberella zeae*, the management of inoculum in individual fields has little or no impact on the regional epidemics of FHB [226,239]. However, among the practices affecting the occurrence of *Fusarium* disease, crop rotation is critical. It is commonly accepted that in cereals grown in monoculture or followed by alternative crops, the potential hosts of *Fusarium* pathogens are at a greater risk of fusariosis and also grain contamination can be related to different amounts of crop debris left on the soil [240]. *Fusarium* spp. population, involved in FHB, is characterized by a large variability and complexity according to location and type of substrate (weeds, crop residues, soil and residual ears) [241]. Evidence that debris of previous crops plays an important role in *Fusarium* infection is suggested by the observation that FHB spreads at the highest values when maize and wheat are adjacent or previous crop compared to a non-host such as soybean, [242].

### 5.2. Cultivar Selection

The use of cereal cultivars resistant to *Fusarium* disease can represent a valid tool to reduce mycotoxin occurrence and the right choice of cultivar is of primary importance, in particular for small cereals. Selection of cultivars in small cereals should take into account the constitutive resistance to FHB that can include: plant height [243], flowering type [244] and time [245], resistance to lodging [246] and trait loci for resistance to *Fusarium* disease. Recently, proteomic and transcriptomic analysis in wheat revealed that in FHB susceptible genotypes, *F. graminearum* infection is related to the delay of defense mechanism activation and that the pathogen take advantage of susceptibility factors to create an appropriate environment for its development [247,248]. FHB resistance is a quantitative trait controlled by multiple genes characterized by considerable variation [249]. Different resistance traits to FHB can be distinguished: the first related to prevent the initial infection (type I) [250], the second operating against fungal spread (type II) [251], a third related to the ability to resist kernel infection (type III) [252], and other two types including tolerance to infection (type IV) [253] or resistance to DON accumulation (type V) [254]. About type V toxin resistance two mechanisms have been proposed: V-1, metabolic transformation of DON to less toxic glucosylated-compounds and V-2, inhibition of trichothecene biosynthesis. [255] Recently, a study on wheat cultivars with different degrees of FHB resistance reported that DON contamination levels did not increased consistently with the concomitant increase of disease incidence; moreover, DON levels in the most FHB sensitive varieties were not necessarily high [256].

In maize, the control strategy for ear rot can be implemented with the use of genetically resistant hybrids with traits unfavourable for fungal colonization and mycotoxin biosynthesis, but at our knowledge few *Fusarium* resistant hybrids has been recently commercialized. The genetic resistance to GER and FER, appear complex with many clustered quantitative trait loci (QTLs) that shown a possible pleiotropic effects on both disease resistance traits and mycotoxin accumulation [257,258,259]. These traits can involve grain hardness [260], the season length of hybrids [261], the physicochemical parameters (pH, a_w_) of grains [262] the nutritional content of kernels [263] or accumulation of antiphenolic compounds [264]. Ear rot in maize can also be decreased by using Bt-maize, which limits corn-borer insect activity, disease occurrence and fumonisin contamination [265].

### 5.3. Planting and Weed Management

Management of planting date, with emphasis on early planting, was demonstrated to be important for *Fusarium* disease control in both wheat and maize cultivars [30,266]. The sowing date and the accurate choice of cereal varieties and maize hybrid, referred to the length of cultural cycle, determine the environmental conditions to which the crop is exposed during silking and grain filling to *Fusarium* inoculum and infection. Therefore, the right agronomic choices could be advantageous with respect to reducing fungal development and toxinogenesis [267]. Field experiments carried out to compare hybrids with different maturity revealed that the cultivation of the early maturing hybrids resulted in a reduced zearalenone contamination related to the conditions in which ripening occurs [260]. In planting management, optimal seed density varies among hybrids, and it is important to avoid the plant-to-plant competition for light, nutrients and water. This phenomenon was extensively investigated in maize and high density was demonstrated to cause a clear yield reduction in drought conditions [268] and a significant increase in fumonisin contamination. Moreover, an increment in plant density of 26% (from 65,000 to 82,000 plants·ha^−1^) was observed to determine higher values of ear rot severity (+43%) and fumonisin content (+153%) [269]. However, other studies reported any change in mycotoxin reduction lowering seeding density (from 98,800 to 49,400 plants·ha^−1^) [221].

Competition and interference between crop and weeds for water, nutrients and sunlight may predispose plant to be more susceptible to the effect of other stressors (shade avoidance ), therefore weed management is necessary to alleviate plant stress and improve crop production [187]. The impact of weeds on the development of *Fusarium* epidemics has also been correlated with their role as a source of inoculum [270,271]; indeed, *Fusarium* spp. have been isolated from a wide range of grasses [270], and a high weed density has been shown to increase FHB disease [272]. Regardless of the positive effects due to a decrease in inoculum, the activity of herbicides such as glyphosate can alter the soil ecosystem through a direct effect on various components of the soil microflora, and can potentially increasing the pathogen population [273]. The effect of weed control with herbicides on *Fusarium* disease is difficult to predict because a significant increase in disease severity has been associated with the wide-spread application of these chemicals. In particular, herbicides are known to predispose plants to specific diseases [274]. Although largely debated [275], the activity of glyphosate weed control in predisposing plants to *Fusarium* disease by impairing plant defenses has been demonstrated [276] in both wheat and maize [277,278]. However, other studies reported that glyphosate had no significant effect on the FHB index and DON content in wheat and barley [279].

### 5.4. Irrigation and Fertilization Regimes

Drought and heat stress can influence *Fusarium* disease occurrence and mycotoxin production, with particular emphasis about fumonisin in maize due to prolonged drought conditions. Wheat and small cereals are subjected to few events of heat and drought in their cultivation areas; however, because irrigation can be required, an increment in FHB severity can represent a collateral effect of the increased moisture [280]. Therefore, when irrigation is required, it should be avoided during anthesis and early grain filling periods [281] especially with regard to DON and ZEA accumulation [282]. In contrast, maize, which requires a higher temperature, frequently encounters these abiotic stress conditions. The mitigation of drought stress by irrigation improves maize yield performance [221] and irrigation has been reported to reduce *F. verticillioides* infection and fumonisin accumulation in maize [283], anyway, some authors did not report significant effects after irrigation treatment [221]. The benefit derived from this practice could be lost due to incorrect irrigation methods: in fact, maize fields irrigated by overhead sprinklers showed significantly higher levels of fungal colonization and fumonisin contamination compared with those that were not irrigated or were surface irrigated [184]. Nevertheless, the effect of the irrigation system on fungal colonization and mycotoxin accumulation is debated and typically, no significant influence due to the water supply system is reported [284]. Possible explanations for these results include the following: irrigation treatments were carried out in the absence of real drought conditions, other types of stress masked the drought effect, and environmental factors were unfavourable to *Fusarium* development.

Some evidence indicates that FHB can be influenced by fertilization regimes; in this respect, it was reported that FHB infection and DON contamination may be directly correlated with an increase in nitrogen fertilization [285], and this could be attributed to a state of physiological stress of crop plants and to the alteration of the crop canopy structure [286]. Variable responses to nitrogen fertilization were reported for maize, where the *Fusarium* mycotoxin concentration was affected differently by the different fertilization regimes [287]. Not only nitrogen but also the availability of micronutrients strongly affect plant growth, resistance to pathogens and stress responses [288] and can predispose plants to diseases as observed for magnesium deficiencies [289].

### 5.5. Insect Management

An important source of *Fusarium* inoculum is related to the activity of insects, and *Fusarium* species have been isolated from a wide range of insects [290]. In small cereals, aphids are important insect-pests often correlated with FHB severity, but the efficacy of insecticide application seems strongly related to pest pressure. While, prophylactic sprays with insecticides will not enhanced wheat yields in absence of high pest pressure [291], in Indian trials the application at heading significantly improved FHB control, but no data were collected on mycotoxin [292]. Further, in Northern Europe the use of insecticides in cereals showed a low effect, although significant, with the infestation by *F. graminearum* and the consequent mycotoxins [286].

In maize, phytophagous insects represent one of the more important infection pathways for *F. verticillioides* infection and consequently, fumonisin contamination. Bt-maize, as well as insecticide treatment, can decrease FER occurrence by reducing potential inoculum infection and fumonisin accumulation [265]. Due to the role of insects in FER infection, a correlation between the borer control and the reduction in *Fusarium*-mycotoxin levels (trichothecenes, fumonisins, zearalenone and moniliformin) was observed under average climatic conditions [188,293], however no significant differences were observed between different insecticides [294,295]. Recent studies suggested a correlation between ECB and the emerging toxins produced by *Fusarium* sp. of the section Liseola (beauvericin, fusaproliferin, fusaric acid and moniliformin); therefore it is presumable suppose a positive effect of the borer control to reduce these toxins [208]. Finally, being insects a lesser pathway for *F. graminearum* infection, insecticides treatments are usually ineffective on GER incidence, however, there are evidences that a reduction can occur, albeit to a lesser extent, in DON contamination [296].

### 5.6. Chemical and Biological Control

Among the direct control strategies, a broad range of chemicals was assayed against *Fusarium* diseases. Fungicidal treatment applied to small cereals against FHB, at least until anthesis or a few days after anthesis [297], is the agricultural practice that has the greatest benefit for grain yield due to a decrease in disease severity and because of the maintenance of good photosynthetic performance of the cereal crops during grain filling [298]. Azole-group fungicides include metconazole, propiconazole, prothioconazole and tebuconazole that belong to the class of demethylation inhibitors. Significant differences between active ingredients were found [299], probably due to the differential sensitivity of different *Fusarium* species to treatment [300], as well as the tolerance of specific pathogenic strains. Further, crop hybrids exhibit significantly different responses to fungicide treatment [301] and evidences suggest the need of an integrated approach [302]. Azole-group fungicides are the most effective in controlling *Fusarium* spp. reducing DON, emergent toxins and fumonisin levels in wheat and maize grain, respectively [301,303,304]. These fungicides tested *in vitro* minimized T-2 and HT-2 contamination of oats by *F. langsethiae* [305], but there was no significant difference in field trials conducted for spring and winter oat varieties [306]. Although these fungicides counteract FHB and trichothecenes, they do not provide complete control. Not-significant reduction or opposite results were observed with regard to ZEA [282,304] and often their efficacy was strongly influenced by disease pressure [235,304].

Although fungicides can be exploited for disease reduction, the application of fungicides in some cases resulted in a significant increase in mycotoxin contamination as observed with DON [307]. This effect was probably related to an increase in *Fusarium* infection due to the activity of the fungicidal molecules on the other microorganisms present within the wheat ear rather than to a direct effect on mycotoxin production [308]. Nonetheless, evidences indicate that fungal exposure to sub-lethal fungicide concentrations can stimulate mycotoxin production and this aspect should be of great concern in cereal cultivation [309]. In fact, some of these molecules are known to trigger oxidative stress, which promotes mycotoxin biosynthesis in *F. graminearum* and upregulates gene expression in *F. verticillioides* [310]. For this reason it is conceivable to assume that a reduction in fungal inoculum, after the application of a fungicide, could not always correspond to a reduction in mycotoxin contamination.

Another strategy to control *Fusarium* spp. colonization and mycotoxin contamination in cereal crops is based on the application of biological control agents (BCAs) and bioactive plant metabolites, which can help to reduce the use of fungicides. Although BCAs may also prove useful in limiting the survival of pathogenic fungi on cereal residues [311], the main target is the control of infection to reduce mycotoxins. In cereal, the most common modes of action of BCAs on cereal spikes include competition for nutrients, the production of antifungal metabolites and the induction of defense responses [312]. The main bacteria with antagonistic abilities against *Fusarium* include *Bacillus*, *Paenibacillus*, *Pseudomonas* and *Streptomyces* spp. [313,314,315,316]; about fungi, *Clonostachys* and *Trichoderma* spp are considered important beneficial antagonist able to counteract mycotoxigenic *Fusarium* [317,318,319]. *Trichoderma* spp. are probably the most effective fungal BCAs and several studies demonstrated their protective effect and the ability to induce systemic resistance, as reported in wheat against *F. culmorum* and maize against *F. verticillioides* [320,321] associated to the reduction in mycotoxin levels.

A further approach is the potential use of bioactive metabolites such as natural antioxidants and phenolic compounds. Some antioxidants are characterized by effects growth and toxin production in the main mycotoxigenic fungi, including *Fusarium* spp. [322]. The *in vitro* identification of compounds capable of limiting the pathogenic and mycotoxigenic potential of *Fusarium* spp. has been demonstrated against species involved in FHB [322,323] and ear rots [324,325] both with antioxidants, phenolic chemicals or essential oils. However, bioactive compound are generally susceptible to degradation promoted by heat, metals, oxygen, light and free radicals [326], therefore the complexation in a stabilizing molecules, such as β-cyclodextrin [327], could improve the application of this substances against plant pathogen in field.

## 6. Conclusions

*Fusarium* disease occurs in cereals when plants, fungal pathogens and environmental conditions are conducive for infection. *Fusarium* disease incidence under similar environmental and conductive conditions is related to the abundance of inoculum present, such as environmental inoculum (soil and airborne), infected crop debris, weeds and phytophagous insects. While airborne inoculum is difficult to predict, *Fusarium* inoculum on crop debris and weeds, and infections in damaged tissue induced by insects can be reduced. Environmental conditions that favour crop susceptibility to pathogens can be prevented or mitigated through the use of correct crop management practices. GAPs are used in farm and orchard production systems to guarantee food safety, *i.e.*, to ensure that foodstuffs are free of contamination caused by harmful compounds. The full application of GAPs towards toxigenic *Fusarium* species requires an integrated approach to manage all the possible risk factors to prevent mycotoxin contamination. It is difficult to evaluate or predict the contribution of direct and indirect stress factors on *Fusarium* disease and mycotoxin occurrence, mainly because of the differential ability of the players (plants and pathogens) to perceive the physiological or environmental changes as condition of stress; this is further complicated by specific responses of strains and cultivars of the same species. The complexity of these issues therefore makes it necessary to consider an integrated approach for *Fusarium* control in cereals by exploiting practices that, on the one hand avoid conditions that can promote plant infection, on the other hand preserve the wellbeing of the plant through stress mitigation.

## Figures and Tables

**Table 1:** Percentage and level of contamination in different countries and commodities relative to deoxynivalenol.

Country	Cereal	Contamination Range (ppb)	Samples	Incidence (%)	Samples Over Limits	Ref.
Argentina	Maize	n.d.–3600	3246	1.1	+(n.a.)	[46]
Brazil	Wheat	183–2150	150	97	+(3.3)	[47]
Canada	Durum wheat	n.d.–4700	54	75	+(n.a.)	[48]
China	Maize	3.3–834.4	132	77	-	[49]
	Wheat	2.4–1130	672	91.5	-	[50]
Croatia	Maize	215–2942	63	71	+(6%)	[51]
	Wheat	115–278	51	65	-	
Finland	Barley	n.a.–1180	34	82.4	-	[52]
	Oat	n.a.–23,800	31	100	+(32%)	
	Wheat	n.a.–5510	30	96.7	+(23%)	
Italy	Durum wheat	n.d.–14,452	240	76.5	+(n.a.)	[53]
	Maize	3–428	140	21.4	-	[54]
Morocco	Wheat	121–1480	80	5	-	[55]
Poland	Maize	n.d.–90	30	66.6	-	[56]
Sweden	Wheat	n.a.–6460	125	82	+(2.4%)	[57]
Syria	Wheat	9–550	40	22.5	-	[58]
Tanzania	Maize	68–2196	60	63	+(5%)	[59]

Limits are referred to European regulation; n.d.: not detected; n.a.: data not available.

**Table 2:** Percentage and level of contamination in different countries and commodities relative to T-2 and HT-2 toxins.

Country	Cereal	Contamination Range (ppb)	Samples	Incidence (%)	Samples Over Limits	Ref.
Croatia	Maize	5–42 *	63	57	-	[51]
	Wheat	6–18 *	51	25	-	
Finland	Barley	n.a–18.1 *	34	20.6	-	[52]
		n.a.–39.5 **		35.3		
	Oat	n.a.–548 *	31	61.3	+(3.2%)	
		n.a.–1830 **		74.2		
	Wheat	1.4–5.4 *	30	46.7	-	
		3.0–15.9 **		63.3		
Italy	Durum wheat	n.d.–212	340	26.5	+(n.a.)	[53]
UK	Oat	n.a.–2321 *	303	84	+(10%)	[60]
		n.a.–6480 **		79		
Tanzania	Maize	15–25 **	60	25	-	[59]

Limits are referred to European regulation, recommended limits are intended for sum of T-2 and HT-2 toxins; n.d.: not detected; n.a.: data not available; * amount of T-2; ** amount of HT-2.

**Table 3:** Percentage and level of contamination in different countries and commodities relative to zearalenone.

Country	Cereal	Contamination Range (ppb)	Samples	Incidence (%)		Samples Over Limits	Ref.
Argentina	Maize	n.d.–10,000	3246	2.7%		+(n.a.)	[46]
Brazil	Wheat	20.4–233	150	32		+(4%)	[47]
China	Wheat	1.13–3048	180	12.8		+(n.a)	[61]
Croatia	Maize	10–611	63	78		+(6%)	[51]
	Wheat	7–107	51	69		-	
Egypt	Maize	0.8–3.5	50	70		-	[62]
Finland	Barley	n.a.–17	34	5.9		-	[52]
	Oat	n.a.–675	31	41.9		+(3.2%)	
	Wheat	n.a.–234	30	46.7		+(3.3%)	
Italy	Maize	n.d–53	140		0.7	-	[54]
Poland	Maize	n.d.–59.9	30		43.3	-	[56]
Sweden	Wheat	n.d.–678	125		46	+(n.a)	[57]
Syria	Wheat	4.–34	40		25	-	[58]
Tanzania	Maize	73–1464	60		5	+(3.3%)	[59]
Tunisia	Durum wheat	n.d.–560	155		79.3	+(23%)	[63]

Limits are referred to European regulation; n.d.: not detected; n.a.: data not available.

**Table 4:** Percentage and level of contamination in different countries and commodities relative to B_1_ and B_2_ fumonisins.

Country	Cereal	Contamination Range (ppb)	Samples	Incidence (%)	Samples Over Limits	Ref.
Argentina	Durum wheat	0.15–1304 *	40	77	-	[64]
	Maize	n.d.–498,212	3246	97.6	+(n.a.)	[46]
	Wheat	0.16–680 *	135	97	-	[64]
Brazil	Cereal mix	n.d.–1876 *	105	83.8	+(2%)	[65]
	Maize	66–7832 *	232	46.6	+(n.a.)	[66]
China	Maize	n.d.–22,362	146	39.7	+(1.4%)	[67]
	Wheat products	0.3–34.6 *	362	6.4	-	[68]
Croatia	Maize	n.d.–4438	63	90	+(1.6%)	[51]
	Wheat	n.d.–203	51	39	-	
Egypt	Maize	59–1915 *	20	100	-	[62]
Guatemala	Maize	10–17100 *	640	98	+(20%)	[69]
Italy	Maize	n.d.–21007	140	97.8	+(25.6%)	[22]
Poland	Maize	59–1190 *	30	100	-	[56]
Syria	Wheat	n.d.–6 *	40	10	-	[58]
South Africa	Maize	10–33,260	288	30	+(16.6%)	[70]
Tanzania	Maize	16–18184 *	60	73	+(15%)	[59]

Limits are referred to European regulation, recommended limits are intended for sum of B_1_ and B_2_ fumonisin; n.d.: not detected; n.a.: data not available; * amount of B_1_ fumonisin.

**Table 5:** Limits relate to human consumption according to European Commission.

**Deoxynivalenol in Food** [89]	
**Commodity**	**Maximum Level (ppb)**
Unprocessed cereals (excluding durum wheat, oats and maize)	1250
Unprocessed durum wheat and oats	1750
Unprocessed maize	1750
Cereals intended for direct human consumption, cereal flour, bran and germ as end product marketed for direct human consumption	750
**T-2 and HT-2 in Food** [93]	
**Commodity**	**Maximum Level Sum of T-2 and HT-2 (ppb)**
Barley (including malting barley) and maize	200
Oats (with husk)	1000
Wheat, rye and other cereals	100
Oats for direct human consumption	200
Maize for direct human consumption	100
Other cereals for direct human consumption	50
**Zearalenone in Food** [89]	
**Commodity**	**Maximum Level (ppb)**
Unprocessed cereals other than maize	100
Unprocessed maize	350
Cereals intended for direct human consumption, cereal flour, bran and germ as end product for direct human consumption	75
Maize intended for direct human consumption, maize based snacks and maize based breakfast cereals	100
**Fumonisin in Food** [89]	
**Commodity**	**Maximum Level Sum of B_1_ and B_2_ (ppb)**
Unprocessed maize	4000
Maize intended for direct human consumption	1000
Maize based breakfast cereals and maize based snacks (a)	800

**Table 6:** Limits relate to cereals intended for animal feed according to European Commission.

**Deoxynivalenol in Feedstuff [155]**	
**Commodity Intended for Animal Feed**	**Guidance Value (ppm)**
Cereals and cereal products with the exception of maize by-products	8
Maize by-products	12
Complementary and complete feedingstuff	5
-exception for pigs	0.9
-exception for calves (<4 months), lambs and kids	2
**T-2 and HT-2 in Feedstuff [156]**	
**Commodity Intended for Animal Feed**	**Indicative Levels Sum of T-2 and HT-2 (ppm)**
Oat milling products (husks)	2
Other cereal products	0.5
Compound feed, with the exception of feed for cats	0.25
**Zearalenone in Feedstuff [155]**	
**Commodity Intended for Animal Feed**	**Guidance Value (ppm)**
Cereals and cereal products with the exception of maize by-products	2
Maize by-products	3
Complementary and complete feedingstuff for	
-piglets and gilts	0.1
-sows and fattening pigs	0.25
-calves, dairy cattle, sheep and goats	0.5
**Fumonisin in Feedstuff [155]**	
**Commodity Intended For Animal Feed**	**Guidance Value Sum of B_1_ and B_2_ (ppm)**
Maize and maize products	60
Complementary and complete feedingstuff for	5
-pigs, horses, rabbits and pet animals -poultry, calves (<4 months), lambs and kids	20
-adult ruminants (>4 months) and mink	50

**Table 7:** Importance of GAPs for mycotoxins control.

Practice	Small Cereal	Maize	
	DON, T-2 and HT-2	Fumonisin	DON and ZEA
**Soil tillage**	VH	L	S
**Crop rotation**	VH	L	S
**Hybrid selection**	H	S	VH
**Planting date**	L	H	VH
**Seed density**	L	S	S
**Weeding**	S	L	L
**Irrigation**	L	S	L
**Balanced fertilization**	S	S	S
**Insecticide treatment**	L	VH	L
**Fungicide treatment**	H	L	L
**Harvest time**	S	H	H

VH (Very High): Extremely important measure for the systemic nature and the remarkable effectiveness in reducing contamination; H (High): Frequently effective measure able to significantly reduce contamination; S (Significant): Often effective measure when it is accompanied by other very effective practices; L (Low): Sometimes effective measure or with reduced effect on contamination.
